# Temporary Anti-Corrosive Double Layer on Zinc Substrate Based on Chitosan Hydrogel and Epoxy Resin

**DOI:** 10.3390/gels9050361

**Published:** 2023-04-25

**Authors:** Tamara-Rita Ovari, Árpád Ferenc Szőke, Gabriel Katona, Gabriella Stefánia Szabó, Liana Maria Muresan

**Affiliations:** 1 Department of Chemical Engineering, Research Center in Electrochemistry and Non-Conventional Materials, Faculty of Chemistry and Chemical Engineering, Babeş-Bolyai University, RO-400028 Cluj-Napoca, Romania; tamara.ovari@ubbcluj.ro (T.-R.O.); liana.muresan@ubbcluj.ro (L.M.M.); 2 Department of Chemistry and Chemical Engineering of the Hungarian Line, Faculty of Chemistry and Chemical Engineering, Babeş-Bolyai University, RO-400028 Cluj-Napoca, Romania; arpad.szoke@ubbcluj.ro (Á.F.S.); gabriel.katona@ubbcluj.ro (G.K.)

**Keywords:** anti-corrosive protection, chitosan/epoxy double layer, chitosan hydrogel, selective peel-off, temporary coatings, zinc

## Abstract

In practice, metal structures are frequently transported or stored before being used. Even in such circumstances, the corrosion process caused by environmental factors (moisture, salty air, etc.) can occur quite easily. To avoid this, metal surfaces can be protected with temporary coatings. The objective of this research was to develop coatings that exhibit effective protective characteristics while also allowing for easy removal, if required. Novel, chitosan/epoxy double layers were prepared on zinc by dip-coating to obtain temporary tailor-made and peelable-on-demand, anti-corrosive coatings. Chitosan hydrogel fulfills the role of a primer that acts as an intermediary between the zinc substrate and the epoxy film to obtain better adhesion and specialization. The resulting coatings were characterized using electrochemical impedance spectroscopy, contact angle measurements, Raman spectroscopy, and scanning electron microscopy. The impedance of the bare zinc was increased by three orders of magnitude when the protective coatings were applied, proving efficient anti-corrosive protection. The chitosan sublayer improved the adhesion of the protective epoxy coating. The structural integrity and absolute impedance of the protective layers were conserved in both basic and neutral environments. However, after fulfilling its lifespan, the chitosan/epoxy double-layered coating could be removed after treatment with a mild acid without damaging the substrate. This was because of the hydrophilic properties of the epoxy layer, as well as the tendency of chitosan to swell in acidic conditions.

## 1. Introduction

Corrosion, defined as the oxidation of a metal due to exposure to an aggressive environmental medium such as salt water, moisture, acidic rain, or UV light, is an ancient problem. Unfortunately, this phenomenon occurs even when the duration of the exposure is short (a few days, weeks, or months), as is the case in the shipping or storage of metallic objects. Painting provides a permanent and unremovable coating with good anti-corrosive properties; however, in the case of metallic parts or equipment that require further assembly, a removable protective layer is more desirable during shipping and storage. Therefore, temporary coatings that can be removed from the surface without damaging the substrate gained importance. Their temporary nature refers to a well-defined service period after which the surface can be liberated by stripping away the coating in specific conditions. Such coatings are used for several applications, including protection from corrosion and mechanical damage, optics, food packaging, antimicrobial applications, cosmetics, and others [1]. Temporary treatment can be used on several substrates, such as ceramics, glass, plastics, and foodstuff; however, metallic surfaces remain one of the most important, and our interest is mainly focused on the anti-corrosion use of such peelable coatings.

It is worth mentioning that, nowadays, there is an increasing interest in multiple functionalities of surface coverings (e.g., flame- and fire-retardant, antimicrobial, self-healing, self-cleaning, and anti-fouling coatings) [2]. Previous studies have been conducted regarding “peelable coatings” with some notable USA patents granted in the 20th century to solve such corrosion problems using a mixture of silicone and titanium dioxide [3] and a mixture of polyvinyl esters, polyacrylates, and polymethacrylates [4]. Furthermore, in the recent literature, a review regarding the peelable coatings [1] summarized the main fields of application and the used materials. In the aforementioned paper, the materials used for anti-corrosion coatings were mainly vinyl polymers and aromatic corrosion inhibitors. The development of mono- and bilayer temporary coatings [5] based on waterborne polyurethane resin applied on stainless steel plates by spray coating has also been reported. Self-assembled layers of phosphates/silanes/thiols were applied as temporary protective films on magnesium alloys [6]. Modifying the adhesion of the first layer in a double-layered system can also lead to the production of a peelable coating [7].

Epoxy-based coatings (EPs) are widely used for the anti-corrosive protection of different metals due to their superior protective properties and ease of application/modification with additives [8]. They also possess characteristics such as outstanding processability, excellent chemical resistance, good insulating properties, and good compatibility with heterogeneous materials in modified coatings. These include materials such as SiO_2_, Zn, Fe_2_O_3_, and halloysite [9], as well as TiO_2_ [10]. EP coatings are relatively hydrophilic because in their cured networks they contain hydroxyl groups, which lead to poor resistance in humid conditions [11]. As such, they can channel aqueous solutions to an underlying layer or substrate.

Chitosan (Chit) is a polysaccharide widely used and studied for several applications in medicine [12] (development of antibacterial agents and wound healing [13]), agriculture, packaging, electroanalysis, the temporary anti-corrosive protection of zinc [14], the protection of biocompatible magnesium alloys [15], and the treatment of heavy-metal ions [16]. This wide array of applications is possible due to its eco-friendly nature, simple production process, and antimicrobial properties. Chitosan hydrogels, beside the fact that they can be easily obtained, are biocompatible and present bacteria-repelling ability. Due to its loose structure, a coating suitable for controlled release of the active ingredient can be made from it [17].

Native chitosan coatings only have modest anti-corrosive properties, which can be improved by using additives or copolymerization. Chit has very good adhesion on Zn, with peeling effects only appearing after excessive crosslinking. It should be noted that, in neutral and basic conditions, chitosan shows only modest swelling due to water intake. In strong acidic environments, as the amine groups of the polymer molecule get protonated, the swelling of the chitosan should be more pronounced, eventually leading to full dissolution if the environment is sufficiently acidic. This should lead to a loss of adhesion toward the substrate. Previous studies show that Chit coatings are removable without damaging the underlying metal [18].

There is a rich scientific literature regarding the investigation of chitosan- and epoxy-based coatings; however, these are hybrid systems or composites. For instance, it was observed that the addition of chitosan decreased the tensile strength [19], increased the flexibility [20], improved the hardness of an epoxy composite [21], enhanced the anti-corrosion property of epoxy coatings [22], and increased the water absorption of the epoxy-based composite [23]. However, to the best of our knowledge, there are no published data for using them as superposed monolayers formed with dip-coating and specialized for temporary protection during the transport of metals that require further processing or assembly.

In this context, the purpose of this research is to produce a novel, peelable coating that offers protection during transport in marine environments (where the main source of corrosion is exposure to NaCl) but can be removed selectively after fulfilling its purpose. To achieve this objective, the studies were carried out in a model solution of 3 *w*/*w*% NaCl. Bisphenol A epoxy resins and chitosan can potentially interact (due to the presence of amino and hydroxy groups on the surface of the components), resulting in coatings with higher stability. In addition, the epoxy layer could potentially channel small amounts of an acidic environment to the chitosan primer layer, which would lose its adhesion to the zinc due to swelling. The greater stability of the chitosan layer in neutral or basic environments, meanwhile, would result in retained adhesion in these conditions.

As such, in this work, chitosan/epoxy double layers were produced on zinc substrates in an attempt to prepare a transparent, temporary anti-corrosive coating with increased protective properties in aggressive conditions, such as a marine environment. As the adhesion of the chitosan sublayer to the zinc surface can be influenced by acids, these coatings should be removable on demand after exposure to such an environment, without damaging the underlying substrate [5].

## 2. Results and Discussion

### 2.1. Coating Thickness and Adhesion Measurements

The determined thickness of the epoxy monolayers on the zinc substrates was ~25 μm, which is one order of magnitude above the irregularities present on the zinc substrate and points to a complete surface coverage, as proven in our previous studies regarding chitosan- [18] and SiO_2_-based [24] coatings on zinc. The chitosan/epoxy double layers had a thickness of ~43 μm. This suggests an increased thickness of the epoxy component of the bilayer, as the thickness of the chitosan sublayer, was only around 8 μm. It was reported [25] that coatings prepared from the same material, even when they are applied with the same technique but on different surfaces (e.g., Zn or Zn/Chit), present different properties. The coatings’ changed structure and surface polarity can be caused by interaction with the substrates. Considering the two structures, there are free amino and hydroxyl groups that can be involved in two types of hydrogen bonds: one, intramolecular for the formation of layers of different thicknesses, and the second, intermolecular that leads to the formation of the multilayered structure, especially between chitosan and the epoxy polymer [26,27].

**Figure 1:**
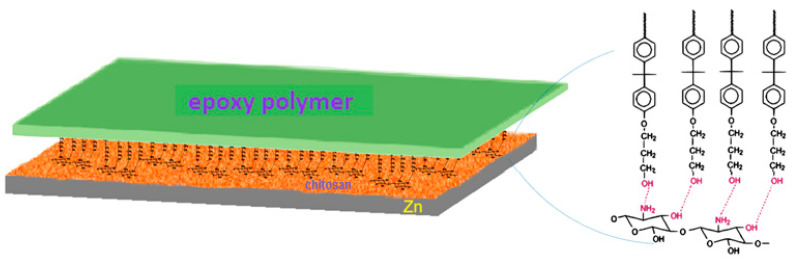
Illustration of the epoxy polymer–chitosan dual layer bonds.

Analyzing the data obtained from the thickness measurements, an increase of thickness can be observed from approximately 33 μm to 43 μm in the case of chitosan deposition followed by the epoxy polymer. The increase in thickness could be explained by the spatial arrangement of the epoxy polymer monolayers, which can be perpendicular to the chitosan surface due to the formation of intermolecular hydrogen bonds, see Figure 1. In this arrangement, the number of hydrogen bonds increases between the two layers resulting in a greater cohesive force, which can be seen in the exfoliation process, when the two layers are removed together.

Raman bands corresponding to the epoxide vibrations in the range of 1230 cm^−1^ to 1280 cm^−1^ were present in the epoxy coating samples, but their intensity is low. This is likely due to the curing reaction involving epoxide ring opening. The Raman peaks observed at around 1100–1200 cm^−1^ and 1608 cm^−1^ are attributed to resin backbone vibrations [28].

In the case of chitosan-coated zinc, no clear peaks were visible where the epoxy shows its characteristic peaks, due to the overlap of multiple peaks attributed to C-H and C-O-C bonds. Moreover, it was reported that, when the chitosan is diluted (as in the case of the aqueous Chit solution used in our case to achieve a thin layer), no separate peaks could be identified in the Raman spectra [29].

**Figure 2:**
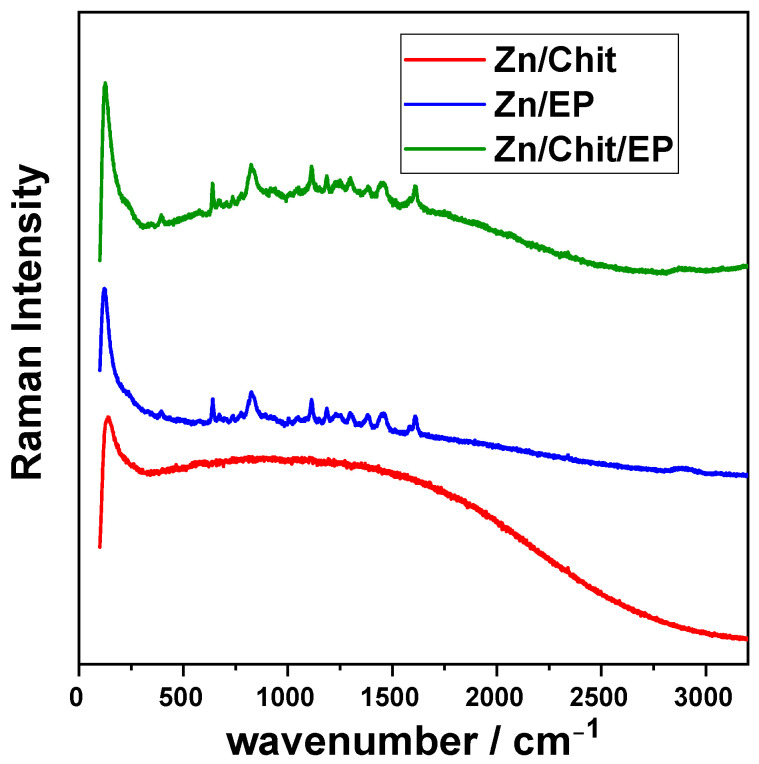
Raman spectra of different coating of zinc substrates.

The Raman spectra (Figure 2) recorded to put in evidence the possible interactions between the two organic layers showed no significant change in peak position, size, or ratio when a chitosan sublayer was present on the zinc substrate under the protective epoxy film. This points to relatively weak interactions between the two layers and a probable lack of covalent bonding. Nevertheless, the affinity between the two organic compounds is sufficiently strong to explain the good mechanic properties of the Chit/EP coating, which is in good accordance with the results obtained for layer thicknesses.

Further, adhesion measurements were carried out in different corrosive media. In a temporary coating, ease of stripping is essential; however, the coating film should not peel off in the studied corrosive media (NaCl in our case), leading to failure during its lifecycle. The acid resistance, alkali resistance, and corrosion resistance of the coating can be evaluated to test its ability to withstand external chemical attack [1].

**Figure 3:**
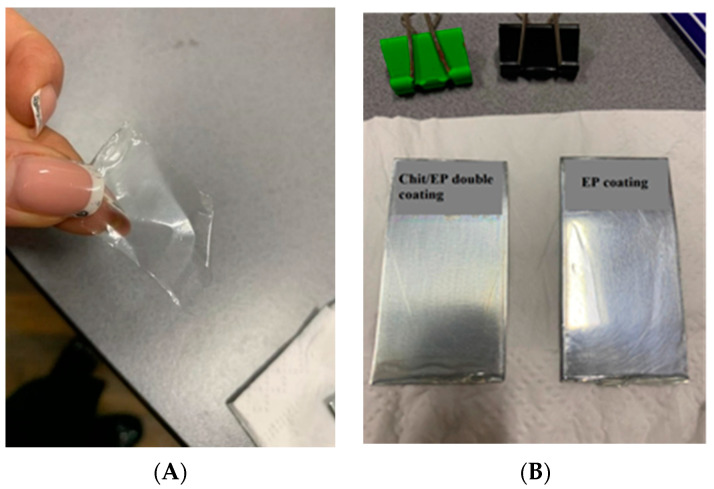
The peeled-off Chit/EP coating (**A**) and the Zn samples coated with Chit/EP and EP layer, respectively (**B**).

Strong wet adhesion inhibits the delamination of the coating during oxygen reduction and hinders the diffusion of the corrosive media at the coating/metal interface [30]. In our case, when the coated samples were soaked in neutral or basic solutions, subsequent visual inspection showed transparent coatings with retained structural integrity. For Chit/EP double layers, adhesion was retained in both NaCl and NaOH solutions, as the layer could not be manually peeled off; however, in the case of the sample soaked in an HCl solution, the coating could be peeled off without damaging the underlying substrate (Figure 3). This can be explained by the epoxy layer channeling the acidic electrolyte toward the chitosan sublayer, causing its swelling and reduced adhesion toward the zinc surface, thus making possible a selective peel-off of the coating. For removable coatings, it is of great importance that the adhesion to the substrate is controlled.

The quantitative adhesion test (Table 1) shows that, in dry conditions, the adhesion of the EP and Chit/EP coatings was similar (~55 and 57%, respectively). Meanwhile, the chitosan showed very good adhesion to the zinc surface with basically no removal. After 2 h of immersion in a NaCl solution, the adhesion of the EP coating in wet conditions dropped to ~27%, while the Chit/EP coating showed only a modest decrease in adhesion from 57% to 51%, showing superior behavior after short-term exposure. Moreover, 24 h after exposure, the EP coating was basically completely removed, while some of the Chit/EP coatings still remained on the surface. These results point to increased adhesion to the zinc substrate when the chitosan sublayer is applied, both in dry conditions and after exposure to a NaCl-containing environment. The adhesive force is essential for the durability of the coating, whereas the cohesive force is essential for easy removal.

### 2.2. Contact Angle and Scanning Electron Microscopy Measurements

**Figure 4:**
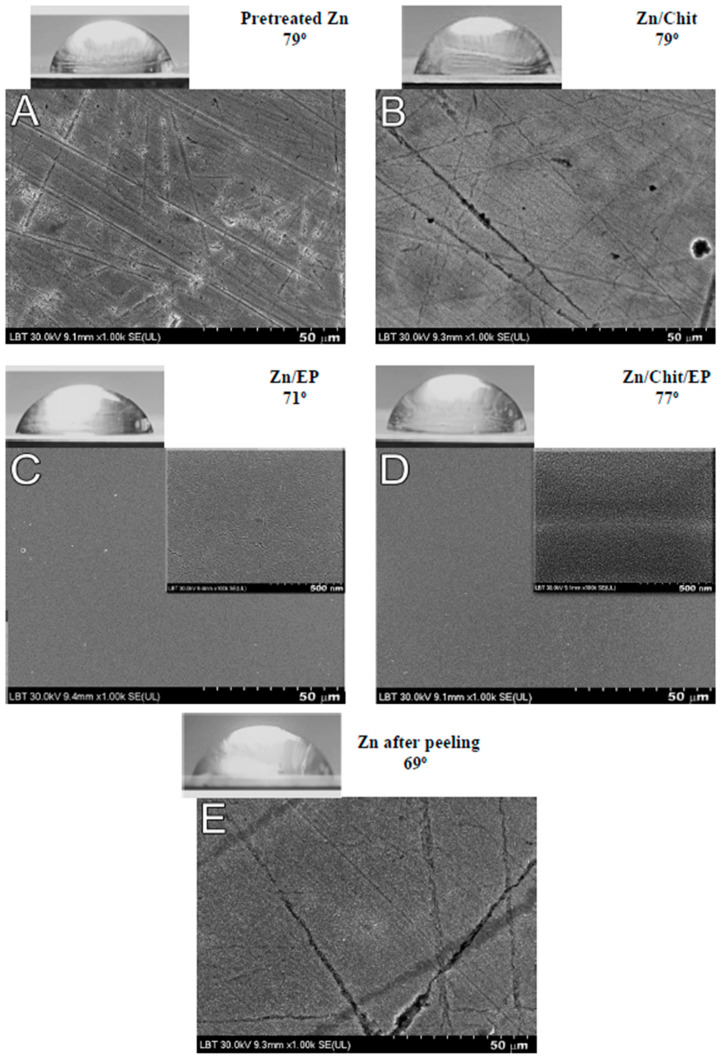
Contact angle measurements and SEM images of pretreated Zn (**A**), Zn/Chit (**B**), Zn/EP (**C**), Zn/Chit/EP (**D**), and Zn after the coating is peeled off (**E**).

To test the hydrophilicity/hydrophobicity of the various surfaces, contact angle measurements were carried out. Compared to other coated systems, a lower initial contact angle was registered in the case of the epoxy layer (71°), underlining its hydrophilic properties (Figure 4). It should be mentioned that the literature shows that the water contact angles measured for epoxy coatings demonstrate very close values [11], pointing to a similar behavior of the coating in different aqueous solutions regardless of the presence of electrolytes. Native chitosan showed a 79° contact angle, which is slightly lower than water contact angles recorded in the literature [31], probably due to the presence of a significant amount of NaCl in the droplet, as high amounts of electrolyte can flow into the chitosan and tend to reduce the contact angle [18]. When chitosan was deposited under the epoxy layer on zinc, this value increased to 77°, suggesting improved structural stability and reduced water intake of the external epoxy layer. The situation of the “sandwich-like coating” differs from that reported for epoxy/chitosan composite coatings, where the incorporation of chitosan in the epoxy matrix resulted in higher hydrophilicity of the material [12]. After the coating was peeled off, the contact angle dropped to 69°, as the zinc substrate may have been covered with some chitosan residue and, possibly, oxidation products, but the quality of the substrate was not significantly affected.

This conclusion is supported by scanning electron microscopy (SEM) measurements carried out to analyze the surface status of the different samples (Figure 4). The native chitosan covers some of the irregularities of the polished zinc surface; in contrast, a uniform coating was obtained when an epoxy layer was deposited on the substrate. Further magnification showed small cracks on the epoxy monolayer, (inset in Figure 4C) which could not be seen when the chitosan sublayer was also present (Figure 4D). This was attributed to improved structural stability. These results are in good accordance with contact angle measurements, where the chitosan underlayer reduced the wettability of the epoxy. After peeling, the zinc surface was regenerated with only low amounts of chitosan residue remaining, covering the major grooves on the metal surface (Figure 4E).

### 2.3. Electrochemical Characterization

First of all, the open circuit potentials (OCPs) were determined for the bare Zn and the Chit-, EP-, and Chit/EP-coated Zn samples in 3 *w*/*w*% NaCl solution. The potential values recorded after 30 min of immersion are presented in Table 2.

It can be seen that the OCP values of coated Zn shifted toward more-positive potentials in comparison to the bare Zn, which means that an ennoblement of the surface can be noticed. As expected, a larger shift was noticed in the case of double-layer-coated zinc.

**Figure 5:**
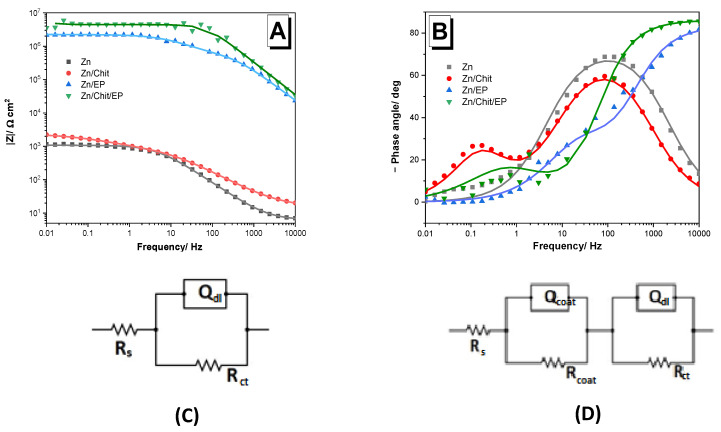
Bode impedance spectra of uncoated Zn and of Zn coated with Chit, EP, and Chit/EP, measured in 3 *w*/*w*% NaCl solution at OCP after 30 min immersion time; (**A**) absolute impedance and (**B**) phase angle plots; the impedance values were normalized to the total surface area of 2 cm^2^. (**C**,**D**) The fitted electrical equivalent circuits.

To determine the protective effect of the Chit, EP, and Chit/EP coatings on the Zn substrate, EIS (electrochemical impedance spectroscopy) measurements were performed on the coated samples at OCP, and the results were compared with those obtained on the bare Zn. The recorded Bode impedance diagrams are compiled in Figure 5.

The good performance of the coatings is reflected by some elements of the Bode plots, such as the absolute impedance ∣Z∣_0.01Hz_, correlated with their barrier properties, and the value of the phase angle at 10 kHz, θ_10kHz_ [32]. Different values of the phase angle suggest different behaviors: a phase angle around −70° suggests that the coatings have a predominant capacitive behavior, −90° corresponds to an ideal capacitor, 0° corresponds to an ideal resistor, and +90° corresponds to an ideal inductor. Values in between may indicate non-ideal, mixed behavior [33].

As can be seen, the Chit coating barely increased the value of the absolute impedance of zinc (ca. 1 kΩ cm^2^), while the EP layer enhanced it by three orders of magnitude (2149 kΩ cm^2^). In the case of the double-layered coating, Chit/EP, this value was even greater (4090 kΩ cm^2^). Moreover, in the last case, the phase angle θ_10kHz_ was also the highest (about −85°).

A comparison with the literature data reported for other protective systems (e.g., the styrene–acrylic coating on steel [30]) led to the conclusion that the impedance modulus of the Zn/Chit/EP coating at low frequencies (~10^6^) was comparable or even higher.

The calculated (Equation (4)) inhibition efficiency IE% value for the Zn/Chit sample was close to 6%. This indicates that, as was expected from previous studies, the Chit coating alone did not offer any significant protection to the metal in the 3 *w*/*w*% NaCl solution, due to its porosity [14,18]. In contrast, the EP and Chit/EP coatings exhibited 99.95%, and 99.97% inhibition efficiency values, respectively. Thus, it can be stated that the Chit/EP bilayer offers better protection than the simple Chit or EP coating.

To better understand this behavior, the experimental impedance data were fitted to electrical equivalent circuits. For the bare, pretreated zinc, the best circuit found was a simple R_s_(Q_dl_R_ct_), where R_s_ is the resistance of the electrolyte solution, while the Q_dl_R_ct_ pair represents the non-ideal charge transfer at the metal/electrolyte interface. In the case of the epoxy-coated systems, both in the presence and absence of a chitosan sublayer, the best fit was obtained with an R_s_(Q_coat_R_coat_)(Q_dl_R_ct_) circuit, where R_s_ is the resistance of the electrolyte, and Q_coat_ and R_coat_ are attributed to the coating, while Q_dl_ and R_ct_ represent the charge transfer at the electric double layer [9]. The same equivalent circuit was used for the chitosan coating.

The electrochemical parameters for the different Zn samples are presented in Table 3. It should be noted that the resistance of the electrolyte solution was negligible in comparison to all other sources of resistance. The polarization resistance, which is the sum of resistances (R_s_ can be omitted), in the case of epoxy layers was three orders of magnitude above those determined for chitosan alone.

Besides bare Zn, the smallest polarization resistance was noticed in the case of Zn/Chit samples. This could be explained by the probability that the electrolyte easily penetrates the porous Chit coating and can reach the coating/metal interface, resulting in a higher capacitance and smaller polarization resistance.

In the case of epoxy-coated samples (Zn/EP), the capacitance values were significantly lower compared to those of the Zn and Zn/Chit systems, and the resistance values were three orders of magnitude higher.

The Zn/Chit/EP coating’s highest anti-corrosive protection can be explained by better physical shielding of the substrate, due to the increased thickness of the coating (see Section 2.1), as well as due to the interactions between the chitosan and epoxy layers, which result in a more stable system.

A similar resistance increase for the double-layer coatings in comparison with the monolayer peelable coatings based on waterborne polyurethane resin was reported in the literature in the case of stainless steel [5].

In addition to the parameters mentioned above, ∣Z∣_0.01Hz_ and θ_10kHz_, the breakpoint frequency (F_b_) plays an increasingly important role in the interpretations of EIS data to analyze the delamination of the organic coatings. The F_b_ is defined as the frequency corresponding to a 45-phase angle at a high-frequency range [34] and can be correlated with the delaminated area A_t_, the total area of the sample A_0_, and a K constant through Equation (1):

(1) $$F_{b}=K\frac{A_{t}}{A_{0}}$$

where K is expressed by Equation (2), which contains the resistivity of the coating ρ, the dielectric constant of electrolyte in the coating ε_0,_ and ε, the vacuum permittivity [34]:

(2) $$K=\frac{1}{2}\cdot \rho \cdot \varepsilon \cdot \varepsilon_{0}$$

A higher value for F_b_ indicates poorer protection [35]. In our case, comparing the F_b_ of Zn/EP (F_b1_ = 137 Hz) and Zn/Chit/EP (F_b2_ = 64 Hz) samples, one can conclude that better protection is provided by the Chit/EP coating. This is in agreement with the calculated electrochemical parameters based on the equivalent electrical circuits.

**Figure 6:**
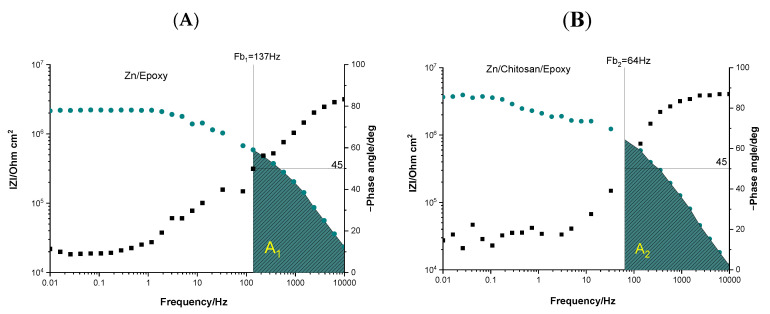
Bode impedance spectra of EP- (**A**) and Chit/EP (**B**)-coated Zn samples, measured in 3 *w*/*w*% NaCl solution after 30 min immersion time at OCP; the impedance values were normalized to the total surface area of 2 cm^2^.

Graphical analysis also can be performed on the Bode diagrams. The area under the horizontal part of the plot at low frequencies, representing the resistive region, can be correlated with the penetration of electrolyte into the coatings’ pores and reflect its barrier properties. The capacitive behavior is related to the inclined part of the curve, and the area underneath corresponds to the capacitive region. The extent of the capacitive region indicates the coating’s performance [32]: a more extensive area shows a more intact and performant coating. In the case of the two abovementioned systems, it can be easily observed (Figure 6) that the capacitive region for the Zn/Chit/EP (A_2_ ≈ 3.77 × 10^9^) is larger than that for the Zn/EP (A_1_ ≈ 2.23 × 10^9^), confirming that the double coating provides better coverage of the zinc substrate.

#### 2.3.1. Influence of the Electrolyte Nature

**Figure 7:**
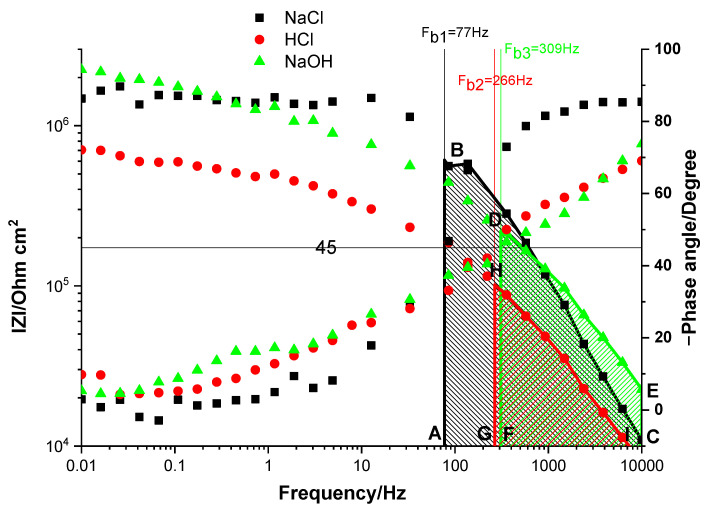
Bode impedance spectra of Chit/EP-coated Zn samples soaked for 2 h in 3 *w*/*w*% NaCl (◼), 0.1 M HCl (●), and 2 M NaOH (▲) solution.

To test the resistance of the coatings in various corrosive electrolytes, the double-layered samples were soaked in 3 *w*/*w*% NaCl, 2 M NaOH, and 1 M HCl solutions, and, from the obtained EIS spectra, the breakpoint frequencies (F_b_) were calculated. To get an interpretation of the coatings’ behavior in different conditions, the obtained Bode spectra were compared (Figure 7). Interestingly, the lowest value of the breakpoint frequency (F_b1_ = 77 Hz) was obtained for the sample soaked in NaCl solution, and the capacitive region (area ABC) in this case had the largest value. This finding points out the protective nature of the coating. After immersion in the acid medium, the breakpoint frequency increased (F_b2_ = 266 Hz), and the lowest area was obtained for the capacitive region (the GHI geometric shape), indicating advanced delamination of the coating. Taking into account that the chitosan is dissolved in acidic mediums and that, by immersion, the aggressive electrolyte can penetrate the coating, the delamination in this case can be easily explained by the adherence loss due to the primer coat’s dissolution. The alkaline medium causes an advanced deterioration of the coating in comparison with the NaCl solution but less than the acidic environment. Although the F_b_ value was lower, as was the capacitive region (marked by DECF), the decrease of ∣Z∣_0.01Hz_ was small compared with that in the NaCl solution. This behavior is probably due to the deterioration of the upper epoxy layer.

#### 2.3.2. Influence of the Immersion Time

**Figure 8:**
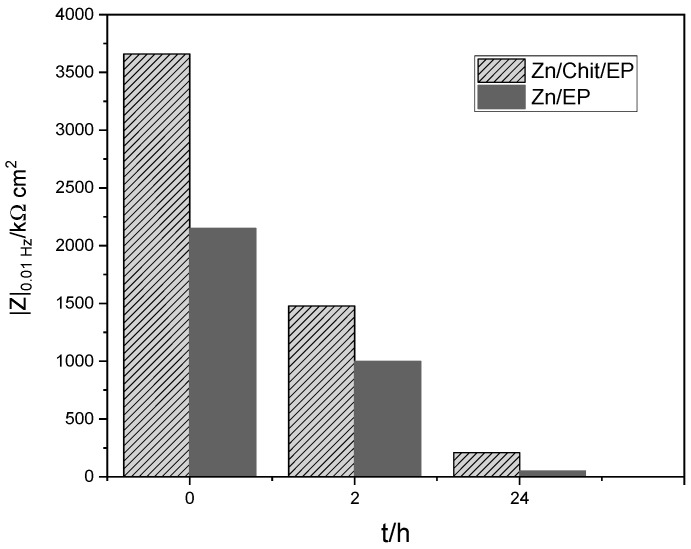
Absolute impedance values for Zn/EP and Zn/Chit/EP determined from Bode spectra as a function of immersion time.

To test the coatings’ resistance over time in highly corrosive NaCl media, Bode impedance spectra were recorded after 2 and 24 h, respectively. As expected, a steady decrease in the absolute impedance after continuous exposure to the corrosive environment was noticed (Figure 8). However, a difference between the various samples was observed. Hence, after 1 day of exposure in 3 *w*/*w*% NaCl solution, both Zn/Chit/EP and Zn/EP samples showed a continuous decrease of their corrosion resistance, but the Zn/Chit/EP presented a much slower degradation. The examination of the coatings’ thickness and their surface will enlighten us to the causes of this behavior (see Section 2.1).

#### 2.3.3. Pseudo-Porosity of the Coatings

The pseudo-porosity of the coatings can be calculated as in previous studies regarding chitosan [14] and titanium nitride [36] coatings using Equation (3):

(3) $$P=\frac{{Rp}_{s}}{Rp}\cdot 10^{-\left|\frac{{∆E}_{corr}}{b_{a}}\right|}\cdot 100\left(\%\right)$$

where P is the pseudo-porosity of the layer, Rp_s_ represents the metal substrate’s polarization resistance, Rp is the coated metal’s polarization resistance, ∆E_corr_ is the difference between the metal’s and the coated sample’s corrosion potential (extracted from linear polarization measurements), and b_a_ is the anodic Tafel coefficient for the substrate. The results are presented in Table 4.

The pseudo-porosity of the Chit/EP system was the lowest, suggesting that the Chit beneath the EP layer sealed the EP layer’s pores, hindering the electrolyte’s access to the Zn substrate, which contributes to the higher corrosion resistance of the double layer.

## 3. Conclusions

Chitosan/epoxy anti-corrosive bilayers were successfully deposited on zinc substrates using the dip-coating method. The chitosan sublayer prepared from hydrogel had a beneficial effect on the anti-corrosive coating. The novel system was characterized by reduced wettability and porosity, improved structural integrity, and increased adhesion especially after exposure to a corrosive environment. All epoxy-based coatings showed good protective properties with an inhibition efficiency of 99.9%. Additionally, the bilayer coatings presented the best corrosion resistance. After soaking in an acidic environment, the Chit/EP film could be peeled off on demand without damaging the underlying substrate.

The impedance of the bare zinc was increased by three orders of magnitude when the protective coatings were applied, proving efficient anti-corrosive protection. The breakpoint frequency (F_b_), defined as the frequency corresponding to a 45-phase angle at a high-frequency range, confirmed the better protection provided by the Chit/Ep coating when compared to one-layer coatings.

In conclusion, chitosan/epoxy bilayers could be a viable, eco-friendly candidate to produce peelable anti-corrosive coatings during the transportation (especially marine transport) of zinc parts. Furthermore, the simplicity of the method should also improve its potential industrial adaptability.

## 4. Materials and Methods

### 4.1. Materials

The Chit was purchased from Aldrich (Darmstadt, Germany) and the EP from MAPEI Romania (Bucharest, Romania). The zinc plates (99% purity) with reduced amounts of Ti and Cu were purchased from Altdepozit (Galati, Romania). NaOH (≥98% purity), HCl (35–38% concentration), and NaCl were purchased from Chempur (Karlsruhe, Germany).

### 4.2. Preparation of Chit and EP Precursors

A 1 *w*/*w*% aqueous Chit solution was prepared [14,18] using medium-viscosity Chit (viscosity: 200–800 cP for 1 *w*/*w*% solid in a 1 *w*/*w*% acetic acid solution). In acidic conditions, chitosan is solubilized by the protonation of amino-groups, and by the dissolution of chitosan flakes, chitosan hydrogel is formed.

The epoxy (bisphenol A epoxy resin) precursor had two components: component A—the epoxy itself and component B—the hardener (a mixture of amine-containing monomers and oligomers). They were mixed in a proportion of 3:1 by mechanical stirring. After 90 min, the EP entered the gel-like form, where it is cured only partially but already loses its workability. To achieve the final cure, the samples were left for 4 days at room temperature to fully cure.

### 4.3. Preparation of the Coated Samples

Before the application of the coating, the zinc plates were pretreated. First, they were polished until mirror-like with abrasive papers of different roughnesses (P1000, P2000, P5000). Particle residue was removed by washing and ultrasonication in isopropanol. Any remaining oxidization on the zinc surface was mostly removed by dipping the substrates for 5 s in 0.1 M HCl solution, before washing them with distilled water. Finally, the zinc plates were wiped with an isopropanol-soaked cotton swab and washed with isopropanol.

An initial chitosan layer was deposited on the cleaned zinc plates using the dip-coating technique at a constant immersion and withdrawal speed of 10 cm/min. The Chit-coated samples were left to dry for 24 h at room temperature. The samples were subsequently coated at a speed of 5 cm/min using the previously prepared epoxy resin precursor.

### 4.4. Characterization of the Coatings

The thickness of the resulting double layers was determined using a BB25 layer-thickness measurer purchased from Trotec. This instrument works by the eddy current method and reads coating thicknesses with a ±1% accuracy. Each sample was measured four times, and an average value was calculated.

The interaction between the chitosan sublayer and the epoxy was studied using Raman spectroscopy on a multilaser confocal Renishaw inVia Reflex Raman spectrometer coupled with an NT-MDT Ntegra Spectra SPM microscope. A 758 nm Renishaw High Power NIR Diode, air-cooled, plasma filter laser line was used with 10 s integration time and 300 mW power.

The adhesion of the coatings was verified using the cross-hatch adhesion test using a Cross Hatch Adhesion Tester from Elcometer and classified according to the ASTM D3359 Classification. The adhesion was determined for dry coatings, as well as coatings immersed in 3 *w*/*w*% NaCl solutions for 2 and 24 h, respectively. In these cases, the cutting occurred right after the layers were immersed and taken out of the corrosive environment. The surface used was a 7 × 7 cm area, from which we determined the percentage of the region that remained after pulling off the applied tape.

The anti-corrosive properties of the coatings were studied using a PARSTAT-2273 single-channel potentiostat (Princeton Applied Research, Oak Ridge, TN, USA) in a three-electrode cell where the coated Zn was the working electrode (2 cm^2^ active area), the Ag/AgCl, KCl_sat_ the reference, and a Pt wire the counter electrode. The corrosive electrolyte solution was 3 *w*/*w*% NaCl, simulating a marine environment. OCPs were recorded for 30 min after immersion (which was sufficient to achieve a stable open circuit potential value for all systems), and all impedance spectra were recorded at OCP with a 10 mV potential perturbation in the 0.01 Hz–10 kHz frequency range.

The inhibition efficiency of each system was approximated using Equation (4) [37]:

(4) $$IE\left(\%\right)=100\times \frac{{{|R}_{p}|}_{s}-{{|R}_{p}|}_{0}}{{{|R}_{p}|}_{s}}$$

where ${{|R}_{p}|}_{0}$ is the polarization resistance of the bare Zn sample, and ${{|R}_{p}|}_{s}$ is the polarization resistance of the coated sample.

The wettability of different systems was assessed through contact angles determined through the sessile drop method, using 3 *w*/*w*% NaCl for the droplets.

The durability of the coatings was verified by soaking the coated zinc samples in different solutions: 0.1 M HCl, 3 *w*/*w*% NaCl, and 2 M NaOH for 2 h before attempting to peel off the protective films.

To examine the surface morphology of the Zn plates (before and after peeling) and the simple (chitosan and epoxy) and double layers (chitosan/epoxy), a Hitachi SU8230 ultra-high resolution scanning electron microscope was used.

## Figures and Tables

**Table 1:** Cross-hatch adhesion test results for coated samples. The percentages show the adhesion of the coatings, followed by the ASTM D3359 Classification. The red rectangles show the 7 × 7 surface from which the adhesion was calculated.

**Immersion Time in** **3 *w*/*w*% NaCl**	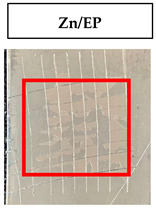	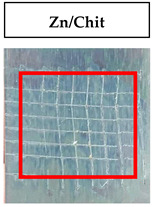	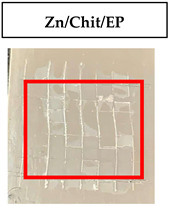
0 h	55.10%, 1B	99.90%, 5B	57.14%, 1B
	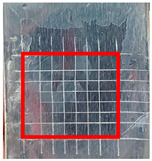	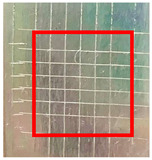	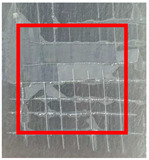
2 h	26.53%, 0B	99.90%, 5B	51.03%, 1B
	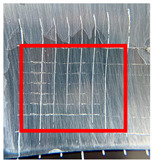	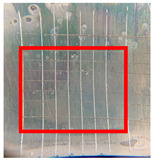	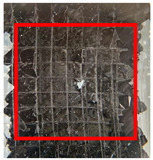
24 h	0%, 0B	99.90%, 5B	24.49%, 0B

**Table 2:** OCP values for various Zn samples coated with Chit, EP, and Chit/EP.

Samples	OCP (V vs. Ag/AgCl/KCl_sat_)
Zn	−0.998
Zn/Chit	−0.981
Zn/EP	−0.979
Zn/Chit/EP	−0.926

**Table 3:** Electrochemical parameter values for Zn, Zn/Chit, Zn/EP, and Zn/Chit/EP samples in 3 *w*/*w*% NaCl solution, calculated by non-linear regression of the impedance data using the equivalent circuit presented in Figure 5C,D (n~0.8).

Sample	R_s_ kΩ cm^2^	Q_coat_ μSs^n^	R_coat_ kΩ cm^2^	Q_dl_ μSs^n^	R_ct_ kΩ cm^2^	R_p_ = R_coat_ + R_ct_ kΩ cm^2^	Chi^2^
Zn	0.01	-	-	56.38	1.08	1.08	6.35 × 10^−3^
Zn/Chit	0.01	865	0.92	1.31	0.23	1.15	8.22 × 10^−4^
Zn/EP	~0.00	0.02	1841	0.0016	383	2224	2.83 × 10^−3^
Zn/Chit/EP	0.44	0.79	875	0.0006	3362	4237	6.71 × 10^−3^

**Table 4:** Pseudo-porosity of the coatings calculated from linear polarization measurements using Equation (3).

Sample	R_p_ (kΩ)	E_corr_ (V)	i_corr_ (µA)	P (%)
Zn	0.43	−1.025	50.70	-
Zn/Chit	0.58	−1.009	37.27	30.18
Zn/EP	1023	−0.932	2.13 × 10^−2^	0.00023
Zn/Chit/EP	4111	−0.891	5.29 × 10^−3^	0.00001

## Data Availability

Authors can confirm that all relevant data are included in the article.

## References

[1] Wagle P.G., Tamboli S.S., More A.P. (2021). Peelable coatings: A review. Prog. Org. Coat..

[2] Dararatana N., Seidi F., Crespy D. (2020). Polymer conjugates for dual functions of reporting and hindering corrosion. Polymer.

[3] Smith H.E., Manor B. (1947). Peelable Protective Coating. U.S. Patent.

[4] Miyata K. (1973). Composition for forming strippable and anti-corrosive film. U.S. Patent.

[5] Gao N., Li J., Zhang W., Ma L., Nwokolo I.K., Liu F., Han E.-H. (2021). Double-layer peelable coating with eminent mechanical properties and anti-permeability. Prog. Org. Coat..

[6] Korrapati V.K., Scharnagl N., Letzig D., Zheludkevich M.L. (2020). Self-assembled layers for the temporary corrosion protection of magnesium-AZ31 alloy. Corros. Sci..

[7] Korrapati V.K., Scharnagl N., Letzig D., Zheludkevich M.L. (2021). Bilayer coatings for temporary and long–term corrosion protection of magnesium–AZ31 alloy. Prog. Org. Coat..

[8] Xiong H., Qi F., Zhao N., Yuan H., Wan P., Liao B., Ouyang X. (2020). Effect of organically modified sepiolite as inorganic nanofiller on the anti-corrosion resistance of epoxy coating. Mater. Lett..

[9] Shi X., Nguyen T.A., Suo Z., Liu Y., Avci R. (2009). Effect of nanoparticles on the anticorrosion and mechanical properties of epoxy coating. Surf. Coat. Technol..

[10] Tan Q., Gao Z., Yan J., Hu W. (2021). Research on superhydrophobicity, corrosion and adhesion properties of composite epoxy-based coating. Mater. Today Commun..

[11] Pourhashem S., Vaezi M.R., Rashidi A., Bagherzadeh M.R. (2017). Distinctive roles of silane coupling agents on the corrosion inhibition performance of graphene oxide in epoxy coatings. Prog. Org. Coat..

[12] Subhi H., Zulkifli Z.A., Mohamad-Noor S.S., Nurul A.A. (2019). Antibacterial properties of gypsum-based chitosan against Streptococcus mutans. Mater. Lett..

[13] Sharma B., Malik P., Jain P. (2018). Biopolymer reinforced nanocomposites: A comprehensive review. Mater. Today Commun..

[14] Szőke F., Szabó G.S., Hórvölgyi Z., Albert E., Végh A.G., Zimányi L., Muresan L.M. (2020). Accumulation of 2-Acetylamino-5-mercapto-1,3,4-thiadiazole in chitosan coatings for improved anticorrosive effect on zinc. Int. J. Biol. Macromol..

[15] Pei Y., Zhang G., Zhang C., Wang J., Hang R., Yao X., Zhang X. (2019). Corrosion resistance, anticoagulant and antibacterial properties of surface-functionalized magnesium alloys. Mater. Lett..

[16] Zhang F., Wang B., Jie P., Zhu J., Cheng F. (2021). Preparation of chitosan/lignosulfonate for effectively removing Pb(II) in water. Polymer.

[17] del Olmo J.A., Pérez-Álvarez L., Martínez V.S., Cid S.B., Ruiz-Rubio L., González R.P., Vilas-Vilela J.L., Alonso J.M. (2023). Multifunctional antibacterial chitosan-based hydrogel coatings on Ti6Al4V biomaterial for biomedical implant applications. Int. J. Biol. Macromol..

[18] Szőke F., Szabó G., Simó Z., Hórvölgyi Z., Albert E., Végh A.G., Zimányi L., Muresan L.M. (2019). Chitosan coatings ionically cross-linked with ammonium paratungstate as anticorrosive coatings for zinc. Eur. Polym. J..

[19] Selvam V., Kumara M.S.C., Vadivel M. (2013). Mechanical properties of epoxy/chitosan biocomposites. Int. J. Chem. Sci..

[20] Jabeen S., Saeed S., Kausar A., Muhammad B., Gul S., Farooq M. (2016). Influence of chitosan and epoxy cross-linking on physical properties of binary blends. Int. J. Polym. Anal. Charact..

[21] Ahmad B., Ashfaq M., Joy A., Carlos Z.A., Sudheer M. (2017). Fabrication and characterization of an eco-friendly biodegradable epoxy/chitosan composites. Am. J. Mater. Sci..

[22] Wonnie Ma I.A., Shafaamri A., Kasi R., Balakrishnan V., Subramaniam R., Arof A.K. (2017). Anticorrosion properties of epoxy-nanochitosan nanocomposite coating. Prog. Org. Coat..

[23] Arukalam I.O., Timothy U.J., Madu I.O., Achor J.O. (2021). Improving the Water Barrier and Anticorrosion Performances of Epoxy-Chitosan Coatings via Silane Modification. J. Bio-Tribo-Corros..

[24] Ovari T.R., Katona G., Szabo G., Muresan L.M. (2022). Electrochemical Evaluation of the Relationship between the Thermal Treatment and the Protective Properties of Thin Silica Coatings on Zinc Substrates. Stud. UBB Chem..

[25] Márton P., Albert E., Nagy N., Tegze B., Szabó G.S., Hórvölgyi Z. (2020). Chemically modified chitosan coatings: Wetting and electrochemical studies. Stud. UBB Chem..

[26] Pillai C.K.S., Paul W., Sharma C.P. (2009). Chitin and chitosan polymers: Chemistry, solubility and fiber formation. Prog. Polym. Sci..

[27] Ramdani N., Chrigui M., Wang J., Feng T.-T., He X.-Y., Liu W.-B., Zheng X.-S. (2014). Preparation and properties of chitosan particle-reinforced polybenzoxazine blends. J. Compos. Mater..

[28] Vašková H., Vojtech K. Raman spectroscopy of epoxy resin crosslinking. Proceedings of the Recent Researches in Automatic Control—13th WSEAS Internation Conference on Automatic Control, Modelling and Simulation, ACMOS’11.

[29] Mendelovits A., Prat T., Gonen Y., Rytwo G. (2012). Improved Colorimetric Determination of Chitosan Concentrations by Dye Binding. Appl. Spectrosc..

[30] Song D., Wan H., Tu X., Li W. (2020). A better understanding of failure process of waterborne coating/metal interface evaluated by electrochemical impedance spectroscopy. Prog. Org. Coat..

[31] Pozzo L.D.Y., da Conceição T.F., Spinelli A., Scharnagl N., Pires A.T. (2018). Chitosan coatings crosslinked with genipin for corrosion protection of AZ31 magnesium alloy sheets. Carbohydr. Polym..

[32] Ramezanzadeh B., Niroumandrad S., Ahmadi A., Mahdavian M., Moghadam M.M. (2016). Enhancement of barrier and corrosion protection performance of an epoxy coating through wet transfer of amino functionalized graphene oxide. Corros. Sci..

[33] Ovari T.-R., Katona G., Coros M., Szabó G., Muresan L.M. (2022). Corrosion behaviour of zinc coated with composite silica layers incorporating poly(amidoamine)-modified graphene oxide. J. Solid State Electrochem..

[34] Li Y., Yang Z., Qiu H., Dai Y., Zheng Q., Li J., Yang J. (2014). Self-aligned graphene as anticorrosive barrier in waterborne polyurethane composite coatings. J. Mater. Chem. A.

[35] Ramezanzadeh M., Ramezanzadeh B., Sari M.G., Saeb M.R. (2020). Corrosion resistance of epoxy coating on mild steel through polyamidoamine dendrimer-covalently functionalized graphene oxide nanosheets. J. Ind. Eng. Chem..

[36] Elsener B., Rota A., Böhni H. (1989). Impedance Study on the Corrosion of PVD and CVD Titanium Nitride Coatings. Mater. Sci. Forum.

[37] Carranza M.S.S., Reyes Y.I.A., Gonzales E.C., Arcon D.P., Franco F.C. (2021). Electrochemical and quantum mechanical investi-gation of various small molecule organic compounds as corrosion inhibitors in mild steel. Heliyon.

